# Evaluation of colposcopy after the addition of human papillomavirus testing to the Turkish cervical cancer screening program

**DOI:** 10.1002/cam4.6740

**Published:** 2023-11-23

**Authors:** Ali Can Gunes, Nejat Ozgul, Murat Turkyılmaz, Fatih Kara, Fahriye Unlu, Ali Ayhan, Murat Gultekin

**Affiliations:** ^1^ Mamak State Hospital Department of Obstetrics and Gynecology Ankara Turkey; ^2^ Division of Gynecologic Oncology, Department of Obstetrics and Gynecology Hacettepe University Faculty of Medicine Ankara Turkey; ^3^ Department of Cancer Control Turkish Ministry of Health, Public Health Institute Ankara Turkey; ^4^ Department of Obstetrics and Gynecology Baskent University Faculty of Medicine Ankara Turkey

**Keywords:** cervical cancer, colposcopy, human papillomavirus

## Abstract

**Objective:**

To evaluate colposcopy performance following the human papillomavirus (HPV) DNA screening program in Turkey.

**Methods:**

Women aged 30–65 years are screened for cervical cancer every 5 years, with individuals positive for HPV 16 and/or 18 or other high‐risk HPV types with abnormal cytology referred for colposcopy. Both HPV test and cytology are obtained at the same visit. If HPV is negative, cytology will not be assessed. However, if HPV is positive, both cytology and HPV genotyping will be performed. Colposcopy‐require was defined as HPV 16/18 positivity or abnormal smear results with any hrHPV positivity, and the remaining patients (normal smear with hrHPV positivity other than HPV 16/18) were grouped as colposcopy non‐required. National data on colposcopy outcomes and unnecessary performance rates in February 2018–2019 were evaluated via a questionnaire.

**Results:**

A total of 9808 patients were included, divided based on colposcopy requirement: 5751 (58.6%) patients required colposcopy and 4057 (41.4%) did not. Unnecessary colposcopy was performed on 90.1% of the non‐required group (3657 of 4057 patients). In the colposcopy‐required group, 4455 patients (79.9%) underwent punch biopsy; 3194 (57.1%), endocervical curettage (ECC); and 421 (7.5%), “see and treat” in the non‐required group, the results were 2790 (76.3%), 1957 (53.2%), and 211 (5.7%), respectively. A total of 746 cervical intraepithelial neoplasia (CIN)‐3 isolates were detected, including 702 using existing screening and triage with 94.1% sensitivity (702/746). Multiple biopsies were taken in 69.8% (*n* = 3110) of patients from the colposcopy‐required group and 63.7% (*n* = 1777) from the non‐required group. The ECC samples included 19 cervical cancers and 212 ≥CIN‐3 lesions in the colposcopy‐required group, and four cancers and 41 ≥CIN‐3 lesions in the non‐required group. The proportion of ≥CIN‐3 lesions detected by ECC only was 4.7% (35 of 746 ≥CIN‐3 lesions).

**Conclusion:**

Our results showed high rates of unnecessary colposcopies, and a high percentage of multiple and random punch biopsies and ECC.

## 1 INTRODUCTION

Cervical cancer is the most common gynecological cancer worldwide. According to GLOBOCAN 2018, it constitutes 6.6% of female cancers and 7.5% of all cancer‐related deaths.^1^ Although it is a common and fatal cancer, screening and early detection are particularly useful. Therefore, screening programs play an important role in the prevention of cervical cancer. WHO announced a global call for action to eliminate cervical cancer. Achieving this goal rests on three key pillars and their corresponding targets: 90% vaccination, 70% screening, and 90% treatment. Due to its high performance (high sensitivity and high negative predictive value),^2^ HPV DNA screening starting at 30 years old with 5–10 yearly retesting is recommended.^3^

**FIGURE 1:**
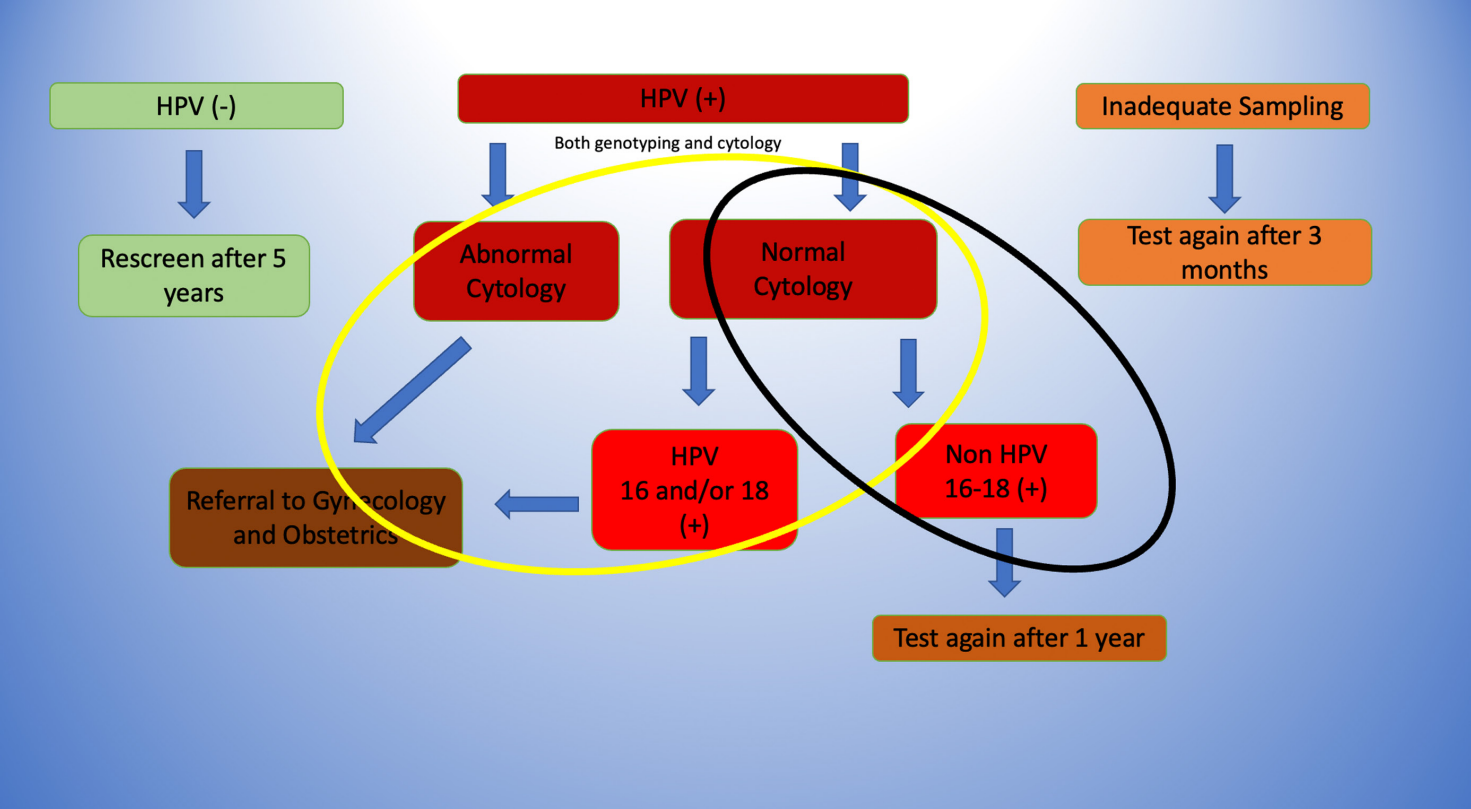
Turkish Ministry of Health's Cervical Cancer Screening Algorithm and method of dividing the colposcopy‐required and colposcopy non‐required groups. Yellow circle is “colposcopy‐required group.” Black circle is “colposcopy non‐required group.” HPV, Human papillomavirus.

According to the new Turkish cervical cancer screening guidelines, women aged 30–65 years have been screened for cervical cancer once every 5 years since 2014.^4^ Both HPV and Pap smear tests are performed during the same visit (one visit double triage strategy),^5^, ^6^ with colposcopy referral for patients with HPV 16 and/or 18 positivity or high‐risk HPV (hrHPV) positivity with abnormal cytology (Figure 1). The double triage strategy has been used to reduce the number of women undergoing colposcopy. Colposcopy is performed in post‐screening diagnostic centers, with at least one in each province of Turkey. Gynecologists who perform colposcopy at these centers receive continuous medical education on this procedure. The Ministry of Health and the Turkish Society for Colposcopy and Cervical Pathology (TRSCCP) have been organizing basic and advanced colposcopy courses 2–3 times per year since 2010.

In 2004, Turkey initiated a Pap smear‐based population‐based cervical screening program. Annual coverage rates were only 1%–2%, and most of them were opportunistic screening. On average, 25,000 women were screened per month since 2004. After implementation of HPV‐based testing, these numbers have increased three times in the first 14 months. We have previously investigated the effectiveness of the primary screening program^6^, ^7^; however, real‐life colposcopy performance after screening has not yet been evaluated. Many countries have tried to establish a standard colposcopic management and organized cervical screening program or improve the quality of clinical effectiveness. While some countries have their national guidelines, the others are on progress or try to follow current accepted American and European guidelines. In countries having own national guidelines, colposcopists are obliged to follow the guideline. However, this is not regulated via a certification program in most of these countries.^8^ For instance, a survey study in England showed that there were great variations among colposcopists about colposcopic biopsy techniques, number of biopsies taken, and rationale for performing a biopsy.^9^ In light of the changing landscape of cervical screening programs, there have been discrepancies between guidelines and real life. This study is the first to prospectively evaluate real‐life data on the colposcopy performance of Turkish gynecologists and especially see how well Turkish colposcopists were following the recommendations.^6^, ^7^

## 2 MATERIALS AND METHODS

### 2.1 Study design

To evaluate the colposcopy performance of gynecologists, the “Colposcopy Evaluation Form” (Appendix 1) was created by the Hacettepe University Obstetrics and Gynecology Department, TRSCCP, and the Ministry of Health's Cancer Department in January 2018. This form was fully completed and forwarded to the Ministry of Health's Cancer Department prospectively every 3 months. The number of questions and the questions themselves were kept to a minimum when the form was inspected. It was intended to assure high compliance in this way. As there is no regulation about quality standards of colposcopy in our Turkey, it was not questioned which guideline colposcopy adequacy was based on. The type of transformation zone (TZ) was not questioned, because the colposcopist might have not got an endocervical speculum to differentiate type 2 and type 3 TZ. Furthermore, all of the colposcopists do not have similar colposcopy devices or sufficient number of other examination instruments (endocervical speculum or every number of Loop Electrosurgical Excision Procedure (LEEP) loops). Consequently, data from 10,944 patients were collected across the country from February 2018 to 2019. A total of 976 patients (8.9%) were excluded from the study because of inconsistencies and flaws in the data; 160 (1.4%) patients were excluded because of loss to follow‐up during colposcopy (refusing biopsy or any other procedures, noncompliance with colposcopy examination, etc.). A total of 9808 patients were evaluated (Table 1). The study sample was an asymptomatic screening population, having HPV positivity. Patients with symptoms (such as cervical mass or abnormal vaginal bleeding), women with hysterectomy for any benign or malign indication and patients who had been treated previously for cervical preinvasive lesions or cervical cancer were not included in this study. In Turkey, asymptomatic screening patients having HPV 16/18 positivity or abnormal smear results with any hrHPV positivity are referred to colposcopy centers^4^ (Figure 1). Since there was no other indication for colposcopy, patients were grouped in terms of colposcopy requirement according to HPV and cytological results.

**TABLE 1:** General epidemiological findings.

Age, mean ± standard deviation	43.1 ± 8.7
	*n* (%)
30–40 years	4571 (46.6)
41–50 years	3043 (31.0)
51–65 years	2194 (22.4)
Cytology results	
ASC‐US	997 (10.2)
ASC‐H	125 (1.3)
LSIL	435 (4.4)
HSIL	14 (0.1)
AGC	38 (0.4)
AIS	1 (0.01)
NILM	3806 (38.8)
Infection	2768 (28.2)
Inadequate	1624 (16.6)
Cytology results (grouped)	
Abnormal	1610 (16.4)
Normal	6574 (67.0)
Inadequate Sampling	1624 (16.6)
HPV genotypes	
16	4062 (29.5)
18	872 (6.3)
31	1132 (8.2)
33	321 (2.3)
35	623 (4.5)
39	677 (4.9)
45	387 (2.8)
51	1270 (9.2)
52	916 (6.7)
56	667 (4.8)
58	654 (4.8)
59	565 (4.1)
68	628 (4.6)
73	28 (0.2)
Others	956 (6.9)
Total	13,758 (100.0)
Colposcopy	9236
Punch biopsy	7245 (78.5)
ECC	5151 (55.6)
See and treat	632 (6.9)
Pathology results	
Normal	4398 (60.4)
CIN‐1	1684 (23.1)
CIN‐2	452 (6.2)
CIN‐3	672 (9.2)
Cervical cancer	74 (1.0)
Pathology results (grouped)	
≤CIN‐2	6534 (89.7)
≥CIN‐3	746 (10.2)
≥CIN‐2	1198 (16.5)

Abbreviations: AGC, atypical glandular cells; AIS, adenocarcinoma in situ; ASC‐H, atypical squamous cell—cannot exclude HSIL; ASC‐US, atypical squamous cell—undetermined significance; CIN, Cervical Intraepithelial Neoplasia; ECC, Endocervical Curettage; HPV, Human papillomavirus; HSIL, high‐grade intraepithelial lesion; LSIL, low‐grade intraepithelial lesion; NILM, negative for intraepithelial lesions or malignancy—normal or infection.

**FIGURE 2:**
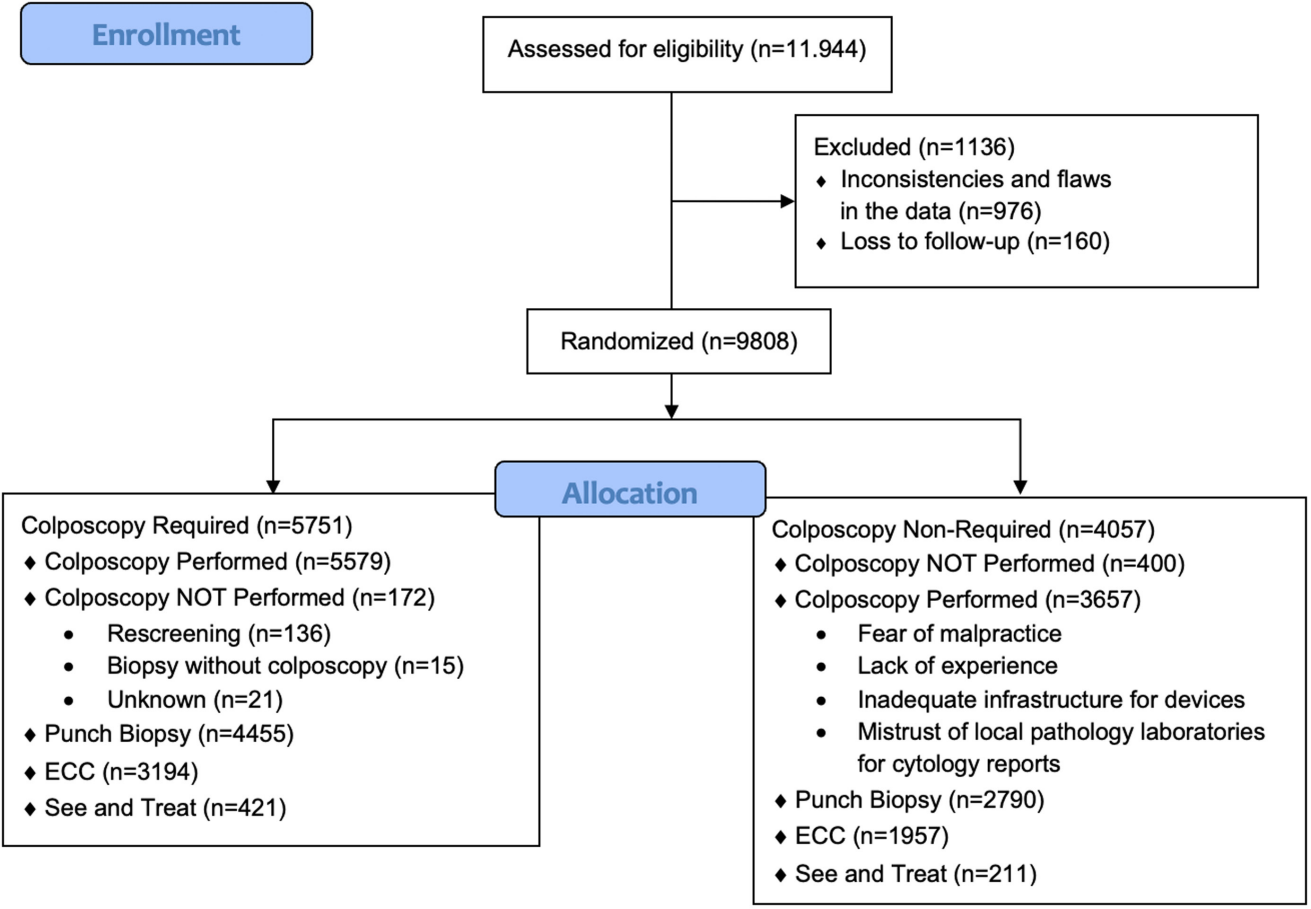
CONSORT flow diagram depicting the study population.

Patients were divided into two groups based on colposcopy requirement. Colposcopy‐require was defined as HPV 16/18 positivity or abnormal smear results with any hrHPV positivity, and the remaining patients were grouped as colposcopy non‐required (Figure 2) (Table 2).

**TABLE 2:** Findings based on colposcopy requirements.

	Colposcopy required, *n* (%)	Colposcopy non‐required, *n* (%)
	5751 (58.6)	4057 (41.4)
Age, mean ± standard deviation	42.6 ± 8.6	43.6 ± 8.9
30–40 years	2806 (48.8)	1769 (43.6)
41–50 years	1771 (30.8)	1278 (31.5)
51–65 years	1174 (20.4)	1010 (24.9)
Colposcopy (9236)	5579 (97)	3657 (90.1)
Adequate colposcopy (6116)	3725 (66.8)	2391 (65.4)
Punch biopsy	4455 (79.9)	2790 (76.3)
Punch biopsy numbers		
1	1345 (30.2)	1013 (36.3)
≥2	3110 (69.8)	1777 (63.7)
ECC	3194 (57.1)	1957 (53.2)
See and treat (LEEP)	421 (7.5)	211 (5.7)
Pathology		
≤CIN‐2	3696 (84.0)	2838 (98.4)
≥CIN‐3	702 (15.9)	44 (1.5)
Cervical cancer	71 (1.6)	3 (0.1)

	Punch Biopsy Numbers		Punch Biopsy Numbers	
	1	≥2	1	≥2
Punch pathology				
≤CIN‐1	983 (29.3)	2368 (70.7)	867 (37.2)	1469 (62.8)
CIN‐2	72 (25.7)	208 (74.3)	40 (31.2)	78 (68.8)
≥CIN‐3	149 (25.9)	427 (74.1)	10 (27.7)	26 (72.3)
Cervical cancer	19 (29.7)	45 (70.3)	1 (50.0)	1 (50.0)
ECC pathology				
≤CIN‐1	2626 (90.2)		1761 (96.4)	
CIN‐2	76 (2.6)		25 (1.4)	
≥CIN‐3	212 (7.2)		41 (2.2)	
Cervical cancer	19 (0.6)		4 (0.2)	
“Only ECC” pathology				
≥CIN‐3	32 (4.6)		3 (6.8)	
Cervical cancer	3 (4.2)		1 (7.7)	

Abbreviations: CIN, cervical intraepithelial neoplasia; ECC, endocervical curettage; LEEP, loop electrocautery excision procedure.

The following variables were evaluated: age; cytology and HPV results; colposcopy rates; punch biopsy, endocervical curettage (ECC), and “see and treat” rates; pathology results; follow‐up findings; treatment modalities. Age was categorized into 30–40, 41–50, and 51–65 year groups.

### 2.2 Cytological evaluation

The Bethesda system was used for cytological evaluation, resulting in the following outcomes: NILM (negative for intraepithelial lesions or malignancy—normal), ASC‐US (atypical squamous cell—undetermined significance), ASC‐H (atypical squamous cell—cannot exclude HSIL), LSIL (low‐grade intraepithelial lesion), HSIL (high‐grade intraepithelial lesion), AGC (atypical glandular cells), other. Cytological results were grouped as normal, inadequate sampling, or abnormal. NILM and infection (any organisms such as bacterial vaginosis, fungal organisms, and trichomonas vaginalis) were deemed normal, and adequate samples in the other categories were deemed abnormal.

### 2.3 HPV DNA analysis

HPV DNA specimen collecting was made by Qiagen HC2 kits. The analysis included 14 oncogenic hrHPV genotypes: HPV 16, 18, 31, 33, 35, 39, 45, 51, 52, 56, 58, 59, 68, 73.^7^ HPV variants other than these 14 oncogenic types were categorized as “other.” None of the patients belongs to the study were HPV‐negative. Patients with multiple HPV types were counted individually.

### 2.4 Outcomes

Punch biopsy numbers were evaluated and divided into two groups: 1 or ≥2. All ECC samples were performed via curette, and endocervical brush was not used. All of the “see and treat” patients underwent loop electrocautery excision procedure (LEEP) during the same colposcopic examination. Patients diagnosed exclusively by ECC were described as “only ECC.” “Pathology results” were all histopathologic diagnosis. The highest cellular abnormality detected from punch biopsy, ECC, or “see and treat” was used as the pathology result and arranged into groups. All benign results (polyp, infection, cervicitis) were included in the normal group. Normal, CIN‐1, and CIN‐2 patients were included in the group ≤CIN‐2, CIN‐3, and cancers into the ≥CIN‐3 group. LEEPs after punch biopsy result, and “see and treat” were evaluated separately. The type of hysterectomy (radical or simple) used has not been described.

### 2.5 Ethical approval and statistical analysis

The study protocol was approved by the Hacettepe University Non‐Interventional Clinical Research Ethics Committee with the approval number GO 20/60. Informed consent was not required for the study.

The data were analyzed using the Statistical Package for the Social Sciences (SPSS) 23.0 (IBM Corp.). Using SPSS 23.0, cross tables were created for epidemiological information, descriptive statistics were prepared.

## 3 RESULTS

### 3.1 General epidemiology

The mean patient age was 43.1 ± 8.7 years, with 46.6% (*n* = 4571) aged 30–40 years, 31.0% aged 41–50 years, and 22.4% (n = 2194) aged 51–65 years (Table 1). Among the 9808 patients, cytology results were normal in 6574 (67.0%), 1624 patients had inadequate samples (16.6%), and the remaining 1610 (16.4%) had abnormal results. Abnormal cytology samples included ASC‐US in 997 (10.2%), LSIL in 435 (4.4%), ASC‐H in 125 (1.3%), AGC in 38 (0.4%), HSIL in 14 (0.1%), and AIS in 1 (0.01%) patient. While HPV 16 and HPV 18 were detected in 4062 (29.5%) and 872 (6.3%) patients, respectively; HPV 51 and HPV 31 were detected in 1270 (9.2%) and 1132 (8.2%) patients, respectively (Table 1).

Punch biopsy, ECC, “see and treat,” and the range of pathology results are shown in Table 1. Punch biopsy was conducted in 7245 (78.5%) patients, ECC in 5151 (55.6%), and “see and treat” in 632 (6.9%) patients. Evaluation of the final pathology revealed that 4398 (60.4%) of the samples were normal, 1684 (23.1%) had CIN‐1, 452 (6.2%) had CIN‐2, 672 (9.2%) had CIN‐3, and 74 (1.0%) had cervical cancer.

### 3.2 Findings based on colposcopy requirements

Of the 9808 evaluated patients, 9236 underwent colposcopy. Colposcopy was adequate in 6116 (66.2%) patients, and the remaining 3120 were deemed to have undergone inadequate colposcopy (33.8%). As shown in Table 2, the 9808 patients were divided into two groups based on colposcopy requirement: 5751 (58.6%) patients required colposcopy and 4057 (41.4%) did not. The age distributions of the required and non‐required groups were similar. Of the 5751 patients who required colposcopy, 5579 (97.0%) underwent colposcopy, whereas 172 (3.0%) did not. Of these 172 patients, 136 (79.1%) underwent rescreening (with smear, HPV, or co‐test) and 15 (8.7%) underwent biopsy without colposcopy. One of these 15 patients had CIN‐1, the other two had CIN‐2, and no cancers were found.

Of the 5579 patients who required and underwent colposcopy, 3725 (66.8%) had an adequate colposcopy, with punch biopsies taken from 4455 (79.9%), ECC performed in 3194 (57.1%), and “see and treat” performed in 421 (7.5%) patients. Of the 4057 patients who did not require colposcopy, 3657 (90.1%) underwent colposcopy. Colposcopy was considered adequate in 2391 (65.4%) and punch biopsy was taken from 2790 (76.3%) patients, while ECC was performed in 1957 (53.2%) patients, and “see and treat” was performed in the remaining 211 patients (5.7%).

The punch biopsy numbers (1 and ≥2) were compared based on the colposcopy requirement. In the group of patients who required colposcopy (5579), one punch biopsy was performed in 1345 (30.2%) patients, whereas ≥2 biopsies were performed in 3110 (69.8%) patients. In the group of patients who did not require colposcopy, one biopsy was obtained from 1013 (36.3%) patients and ≥2 biopsies from 1777 patients (63.7%) (Table 2).

In the group requiring colposcopy, ≥CIN‐3 lesions were found in 702 (15.9%) patients, and cervical cancer was found in 71 (1.6%) patients. In the colposcopy non‐required group, the rates were 44 (1.5%) and 3 (0.1%), respectively. A total of 746 CIN‐3 isolates were detected in the study, with 702 cases detected using the existing screening and triage program, with a sensitivity of 94.1% (702/746).

Forty‐four ≥CIN‐3 lesions detected in the non‐required group were further analyzed. Despite comprising a small proportion of ≥CIN‐3 cases (5.9%), these patients were investigated to determine why there were classed as not requiring colposcopy. Fifteen were found to have inadequate cytology; however, we could not differentiate the reason in the remaining 29 patients. Of the three cancer patients, one had adenocarcinoma (43 years old, HPV 31, 39+, cytology: infection, cancer detected by ECC), one had adenosquamous cancer (41 years old, HPV 51+, inadequate cytology), and the last had microinvasive squamous cancer (54 years old, HPV 45+, inadequate cytology).

### 3.3 Association between punch biopsy numbers and pathology results

Table 2 summarizes the final pathological results with respect to the number of punch and endocervical biopsies. Of the 5751 patients who required colposcopy, 3351 (983 by one biopsy + 2368 by ≥2 biopsies) had ≤CIN‐1 detected by punch biopsy, 280 (72 + 208) had CIN‐2 detected, and of the 576 (149 + 427) where ≥CIN‐3 was detected, 64 had cancer detected. ≥2 punch biopsies were taken in 2368 (70.7%) of the 3351 patients with ≤CIN‐1, 208 (74.3%) of the 280 CIN‐2 patients, 427 (74.1%) of 576 ≥CIN‐3 patients, and 45 (70.3) of 64 cancer patients.

There were 2256 ≤CIN‐1 patients in the non‐required group. CIN‐2 was detected in 118 patients, and ≥CIN‐3 was detected in 36 patients, of which two had cervical cancer. In this group, 1469 (62.8%) of 2256 patients with ≤CIN‐1, 78 (68.8%) of 118 CIN‐2 patients, 26 (72.3%) of 36 ≥ CIN‐3 patients, and 1 (50.0%) of 2 cancers had ≥2 biopsies.

### 3.4 Role of ECC

Of the 9236 patients, 6116 (66.2%) underwent adequate colposcopy, whereas in 3120 (33.8%) patients, colposcopy was inadequate. Transformation zone was visualized in 6096 (99.7%) of 6116 patients who underwent adequate colposcopy, and ECC was performed in 3098 (50.6%). TZ was not visible in 2976 (95.4%) of 3120 patients who underwent inadequate colposcopy, but an ECC was performed in 2053 (65.8%).

ECC was performed in 3194 patients within the colposcopy‐required group (57.1%) compared to 1957 patients within the non‐required group (53.2%). Final pathology reports of these ECC samples found 19 cervical cancers and 212 ≥CIN‐3 lesions in the colposcopy‐required group, and four cancers and 41 ≥CIN‐3 lesions in the non‐required group.

The proportion of ≥CIN‐3 lesions detected by “only ECC” was 4.7% (35 of 746 ≥CIN‐3 lesions) comprising 0.38% of the colposcopies (35 of 9236 colposcopies). The proportion of cervical cancer detected by “only ECC” was 5.4% (4 of 74 cervical cancers) and 0.04% (4 of 9236) of colposcopies. Three of these four patients were in the colposcopy‐required group, while one was in the colposcopy non‐required group. The patient in the colposcopy non‐required group had cervical adenocarcinoma (43 years old, HPV 31, 39+; cytology: infection, TZ: not visible, punch biopsy pathology: normal). The three patients in the colposcopy‐required group are detailed below: one patient with adenocarcinoma (from ECC), 40 years old, HPV 16+, cytology: AGC, TZ: not visible, Punch biopsy pathology: CIN‐1; one patient with squamous cell carcinoma (from ECC), 49 years old, HPV 18+, cytology: infection, TZ: not visible, punch biopsy pathology: CIN‐2; one patient with squamous cell carcinoma (from ECC), 54 years old, HPV 35, 52, 61+, cytology: inadequate sample, TZ: not visible, punch biopsy: not performed, “see and treat” pathology: CIN‐1.

### 3.5 Final treatment according to pathology results

The different management options based on pathology results are shown in Table 3. Follow‐up was recommended for 1182 (87.7%) of the 1348 patients in the CIN‐1 group. Of the 356 patients with CIN‐2, 80 (22.5%) had LEEP after punch results and 84 (23.6%) had cold knife conization, while 165 (46.3%) patients were recommended for follow‐up. ≥CIN‐3 was detected in 641 patients, 498 (77.7%) were treated, and 143 (22.3%) were recommended followed up. Invasive cervical cancer was not detected in any of the 143 patients. There was no follow‐up of the patients with cancer. Among the treatment modalities, cold knife conization was used in 236 ≥ CIN‐3 patients (36.8%), LEEP after punch was used in 110 ≥CIN‐3 patients (17.2%), and hysterectomy was performed in 99 ≥CIN‐3 patients (15.4%).

**TABLE 3:** Follow‐up and treatment rates by final pathology results.

	Follow‐up	LEEP (After punch)	Cold knife conization	Hysterectomy	Cryotherapy	LEEP (see and treat)	Total
Normal, *n* (%)	4398 (100.0)	0 (0.0)	0 (0.0)	0 (0.0)	0 (0.0)	0 (0.0)	4398 (100.0)
CIN‐1, *n* (%)	1182 (87.7)	123 (9.1)	22 (1.6)	7 (0.5)	1 (0.1)	13 (1.0)	1348 (100)
CIN‐2, *n* (%)	165 (46.3)	80 (22.5)	84 (23.6)	6 (1.7)	0 (0.0)	21 (5.9)	356 (100)
≥CIN‐3, *n* (%)	143 (22.3)	110 (17.2)	236 (36.8)	99 (15.4)	1 (0.2)	52 (8.1)	641 (100)

Abbreviation: LEEP, loop electrocautery excision procedure.

## 4 DISCUSSION

Colposcopy is a challenging procedure that plays a crucial role in cervical cancer screening, helping to detect cervical precancers that can be treated. To perform clinical colposcopy satisfactorily, specialized education on anatomy and histopathology is required. In addition, there are global challenges with standardizing terminology, organizing continuous training, implementing quality assurance measures, and creating sufficient infrastructure. In countries without well‐established infrastructure, the sensitivity of colposcopy may be too low, which may result in unnecessary biopsies. Screening with HPV DNA may increase the number of colposcopy referrals dramatically.^10^, ^11^ Due to these challenges, unnecessary colposcopy referrals should be avoided where possible. In our previous study, the rate of referral for colposcopy in Turkey was 1.6%, with an additional 1.2 patients sent to each colposcopy center daily.^6^ While data suggest the Turkish screening program is effective, this is the first prospective study to evaluate the daily practices of Turkish colposcopists.^6^, ^7^

According to the European Federation for Colposcopy (EFC) colposcopy quality standards, colposcopy examination should be performed in all cases prior to treatment for an abnormal cervical screening test^12^, ^13^: The rate was 97% in this study. However, we found a high rate of unnecessary colposcopies that were performed despite not meeting the criteria (90.1%). We could not differentiate the individual reasons for unnecessary colposcopy. However, when we asked several colposcopy experts, the reasons expressed were lack of experience, fear of malpractice, inadequate infrastructure for colposcopy devices (such as low‐quality devices), and mistrust of local pathology laboratories due to inadequate cytology results. Improving colposcopy education, implementing a quality assurance system, and including molecular triage tests (such as p16/Ki‐67 dual‐staining or methylation markers) are necessary to decrease referral rates and to improve adherence to the national guidelines.^14^, ^15^, ^16^

The second finding of this study was the high rate of inadequate cytology. Of the 9808 patients evaluated, 1624 (16.6%) showed inadequate cytology results; the expected proportion of unsatisfactory cytology results in a qualified center is ≤1%.^17^, ^18^ This may be contributed to unnecessary colposcopy, as described above. These high rates need to be examined, and efforts made to reduce them. The guideline has a sensitivity of 94.1% for ≥CIN‐3, on the other hand, 41 CIN‐3 lesions and three cervical cancers were detected in the colposcopy non‐requirement group. The guidelines recommend screening after 3 months in inadequately sampled patients and optionally after 6 months or 1 year in 16–18 HPV‐negative patients with normal cytology, instead of immediate colposcopy.^19^ In this situation, it is expected that some of these missed patients may regress after 6 months and will be identified during follow‐up screening.

Multiple cervical punch biopsies were frequently performed, regardless of effectiveness. However, there was a significant correlation between cervical lesions discovery and the rise in punch biopsies. Punch biopsy results were separated into groups of 1 and ≥2, and the multiple biopsy group had a three times higher detection rate of ≥CIN‐3 lesions. Traditionally, punch biopsies are restricted to lesions with the most serious impression of colposcopy.^20^, ^21^ According to that punch biopsy should be obtained from the worst one among the lesions such as thin aceto‐white epithelium, fine punctation, or dense aceto‐white epithelium. Many studies suggest that multiple biopsies may be beneficial even when colposcopic evaluation reveals no abnormality.^22^, ^23^, ^24^ According to a study by Pretorius et al.^24^ performing multiple biopsies increased sensitivity regardless of experience. However, EFC did not include the average number of punches or the percentage of punch biopsies among their quality standards.^12^, ^13^ A survey of the British Society for Colposcopy and Cervical Pathology‐accredited colposcopists' use of punch biopsy also found significant differences in the average punch biopsy numbers.^9^ American Society for Colposcopy and Cervical Pathology (ASCCP) recommends at least two and up to four targeted colposcopic punch biopsies. Random (nontargeted) punch biopsy is not recommended for low‐risk patients. In high‐risk patients (nonpregnant, 25 years and older, HPV 16/18+, high‐grade cytology, high‐grade colposcopic impression), excisional treatment or multiple targeted biopsies are acceptable.^25^, ^26^ There is still no definitive guidance on punch biopsy number; however, in light of these findings, we suggest taking a minimum of two punch biopsies in patients with colposcopy when punch biopsy is required.

In our study, patient groups with and without colposcopy indications had both similarly high rates of ECC. There are various criteria for ECC during colposcopy, including insufficient colposcopy, ≥CIN‐2 colposcopic findings, and advanced age.^27^, ^28^, ^29^, ^30^ In 2015, Pretorius et al.^31^ reported that when ECC indication was restricted to inadequate colposcopies and/or colposcopies with the impression of a ≥CIN‐2 lesion, a considerable amount of the ≥CIN‐3 lesions that only ECC could detect might be missed. It is important to determine the occult ≥CIN‐3 lesion rate in patients with colposcopy and punch biopsy results of ≤CIN‐2; ECC may be the only way to detect these patients. As the proportion of ≥CIN‐3 lesions detected by “only ECC” was 0.5% of all colposcopies and 7.2% of all ≥CIN‐3, further discussion is required on additional ECCs required to detect this small number of patients and what the ECC indications should be.^31^ In our study, the proportion of only ECC‐detected ≥CIN‐3 was 0.38% (35 of 9236 colposcopies) and 4.7% of all ≥CIN‐3 lesions (35 of 746 ≥CIN‐3 lesions): lower than that reported by Pretorius et al.^31^ However, all our patients were HPV‐positive, and only 50% underwent ECC unlike the 100% in the reference study. We recommend continuing ECC based on the currently accepted indications.

When evaluating the treatment preferences of Turkish gynecologists, we found acceptable rates of follow‐up versus treatment. The majority of CIN‐1 lesions were recommended for follow‐up (87.7%), while 77.7% of ≥CIN‐3 cases were treated. Hysterectomy was almost exclusively used in ≥CIN‐3 cases (15% of ≥CIN‐3 cases), and less than 1–2 percent in CIN‐1 and CIN‐2 cases.

One of the limitations of our study is that the colposcopy evaluation form did not include all colposcopy findings. According to EFC colposcopy quality standards, documentation on whether a TZ (type 1, 2, or 3) is present should be recorded in 100% of cases.^12^ In our study, TZ presence/absence was recorded in 67.6% of patients. Another limitation is the subjectivity of the physicians' definition of adequate colposcopy. There are no national regulations or assessments to determine the adequacy of colposcopy. Cooperation between the TRSCCP and the ministry of health's cancer department is important to coordinate training and quality assessments of the physician/device/institution performing colposcopy.

In conclusion, this is the first study to present real‐life data on the colposcopy performance of Turkish gynecologists. Our results showed high rates of unnecessary colposcopies and a high percentage of multiple and random punch biopsies and ECC. The ministry of health's cancer department and TRSCCP should organize workshops to improve knowledge of colposcopy devices and continue gynecologist training. Quality assurance systems for colposcopy should be implemented, and further studies reviewing the EFC or ASCCP colposcopy quality standards are needed.

## AUTHOR CONTRIBUTIONS

**Ali Can Gunes:** Conceptualization (lead); writing – original draft (lead); writing – review and editing (lead). **Nejat Ozgul:** Methodology (equal); supervision (equal). **Murat Turkyılmaz:** Formal analysis (equal); validation (equal). **Fatih Kara:** Formal analysis (equal); validation (equal). **Fahriye Unlu:** Formal analysis (equal); validation (equal). **Ali Ayhan:** Supervision (equal). **Murat Gultekin:** Conceptualization (equal); supervision (lead); writing – original draft (equal).

## CONFLICT OF INTEREST STATEMENT

The authors declare that they have no conflict of interest.

## ETHICS STATEMENT

The study was approved by the Non‐interventional Clinical Researches Ethics Board of Hacettepe University with the approval number GO 20/60.

## Data Availability

Available.

## APPENDIX 1

### 1.1

CANCER CONTROL DEPARTMENT, MINISTRY of HEALTH (TURKEY) COLPOSCOPY EVALUATION FORM.

ID NO:

NAME SURNAME:

HPV: POZITIVE ( )/NEGATIVE ( ).

HPV TYPE:

CYTOLOGY:

NEXT STEP:

**Table:**

	DATE	RESULT
(A) FOLLOW‐UP WITH SMEAR		
(B) FOLLOW‐UP WITH HPV		
(C) FOLLOW‐UP WITH SMEAR + HPV		

(D) COLPOSCOPY: YES ( )/NO ( ).

**Table:**

(a) Adequate colposcopic evaluation?	YES ( )/NO ( )
(b) Transformation zone was seen?	YES ( )/NO ( )
(c) Punch biopsy?	YES ( )/NO ( )
(d) Punch biopsy number:	1 ( ) 2( ) 3( ) ≥4( )
(e) See and Treat?	YES ( )/NO ( )
(f) ECC:	YES ( )/NO ( )

FINAL PATHOLOGY RESULT:

FINAL TREATMENT METHOD:

(a) Follow‐up.

(b) See and Treat.

(c) LEEP.

(d) Cold Knife Conization.

(e) Hysterectomy.
